# Multiple-Step Melting/Irradiation: A Strategy to Fabricate Thermoplastic Polymers with Improved Mechanical Performance

**DOI:** 10.3390/polym11111812

**Published:** 2019-11-05

**Authors:** Jingxin Zhao, Jiayao Wang, Xiaojun Ding, Yu Gu, Yongjin Li, Jingye Li, Jichun You

**Affiliations:** 1 Shanghai Institute of Applied Physics, Chinese Academy of Sciences, Shanghai 201800, China; 2 University of Chinese Academy of Sciences, Beijing 100049, China; 3 The Education Ministry Key Lab of Resource Chemistry, Shanghai Key Lab of Rare Earth Functional Materials, College of Chemistry and Materials Science, Shanghai Normal University, Shanghai 200234, China; 4 Hangzhou Normal University, Hangzhou 310036, China

**Keywords:** crosslinking, irradiation, PVDF, strength, ductility

## Abstract

To fabricate thermoplastic polymers exhibiting improved ductility without the loss of strength, a novel multiple-step melting/irradiation (MUSMI) strategy was developed by taking poly(vinylidene fluoride)/triallyl isocyanate (PVDF/TAIC) as an example, in which alternate melting and irradiation were adopted and repeated for several times. The initial irradiation with a low dose produced some local crosslinked points (not 3-dimensional network). When the specimen was reheated above the melting temperature, they redistributed in the PVDF matrix, which is an efficient way to avoid the high crosslinking density at certain positions and the disappearance of thermoplastic properties. During the subsequent cooling process, the crosslinked domains in the thermoplastic polymer matrix is expected to play double roles in turning crystal structures for enhancing the ductility without reducing strength. On one hand, they can act as heterogeneous nucleation agents, resulting in higher nucleation density and smaller spherulites; on the other hand, the existence of crosslinked structures restricts the lamellar thickening, accounting for the thinner crystal lamellae. Both smaller spherulites and thinner lamellae contribute to better ductility. At the same time, these local crosslinked points enhance the connectivity of crystal structures (including lamellae and spherulites), which is beneficial to the improvement of strength. Based on the influence of local crosslinked points on the ductility and strength, thermoplastic PVDF with much higher elongation at break and comparable yielding stress (relative to the reference specimen upon strong irradiation only once) was prepared via MUSMI successfully.

## 1. Introduction

In the wide applications of various materials, mechanical performance plays an important role [1,2]. Much attention has been paid to strength and ductility, which can be evaluated by the parameters of yielding stress and elongation at break, respectively. There have been many strategies to enhance the strength or ductility separately [3,4]. The improvement of one parameter, however, always leads to the loss of the other. This is well known as the strength–ductility trade-off effect [5,6]. Different than conventional metals and ceramics, the mechanical performance of polymers is determined by structures in various scales [7,8,9,10]. On one hand, the configuration of polymer chain (the first order structure) produces significant effects on the properties. For instance, the strength was enhanced upon crosslinking poly(ethylene-co-vinyl acetate) random copolymers with dicumyl peroxide (DCP). The results indicated that the mechanical performances were under the control of crosslinking density. Higher density contributes to higher strength but lowers ductility. The resultant mechanical performance dominates its applications, e.g., 3D printing [11,12,13]; on the other hand, the properties of polymers exhibit obvious dependence on the aggregation structures. This is very typical in crystallizable polymers, in which higher crystallinity and bigger spherulites determine the higher yield stress and lower rupture energy [14]. Both crystallinity and crystal structures are related to the nucleation density during crystallization. The results from Jariyavidyanont revealed that the crystallization behaviors can be accelerated by the nucleation effect, resulting in the increase of nucleation density and distinct spherulitic morphologies [15].

Crosslinking is an efficient way to tailor the macroscopic performances of polymer materials, by manipulating the first order structure, as well as the aggregation structure [16,17,18]. For one thing, the connectivity by chemical bonds among polymer chains contributes to the three-dimensional (3D) network and different mechanical performance directly; for another thing, crosslinking produces a considerable effect on the aggregation structures. In the blend of poly(ethylene-co-octene) (POE) and isotactic polypropylene (iPP), Tian and his co-workers found that crosslinking of the former induced the interfacial crystallization of the latter. The crosslinking structures not only enhanced the ability of iPP to maintain the oriented conformation at the interface but also increased the nucleation density significantly [19]. The structures with high crosslinking density, however, always corresponded to thermoset materials, which cannot be shaped for a second time. To prepare thermoplastic crosslinked polymers, both dynamic crosslinking and micro-crosslinking were introduced [20,21,22,23,24,25]. In the former, the blends of poly(vinylidene fluoride) (PVDF) with rubbers (e.g., fluororubber or natural rubber) were dynamically cross-linked, resulting in sea–island or core–shell structures. For instance, Xu et al. prepared thermoplastic vulcanizate based on PVDF and silicone rubber by means of irradiation; in the latter, polymers with low crosslinking density were prepared to balance the mechanical and other properties (e.g., degradation rate). In the strategies discussed above, the strength or ductility suffered from the lower crosslinking density were expected to improve further. 

**Figure 1:**
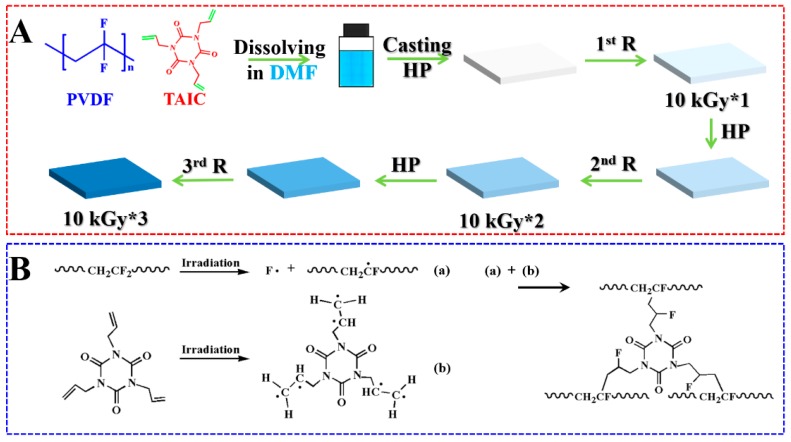
(**A**) The preparation of thermoplastic poly(vinylidene fluoride) (PVDF) with enhanced mechanical performance by multiple-step melting/irradiation (MUSMI). R and HP represent irradiation and hot-press, respectively. (**B**) illustrates the possible reaction mechanism induced by gamma irradiation.

In this work, therefore, a multiple-step melting/irradiation (MUSMI) strategy (Figure 1A) was developed to prepare thermoplastic polymers with improved mechanical performances, by taking poly(vinylidene fluoride)/triallyl isocyanurate (i.e., PVDF/TAIC) as an example. This system has been widely investigated since PVDF and TAIC exhibit excellent miscibility [26,27,28,29]. TAIC can act as the crosslinker to connect PVDF chains, contributing to the local crosslinked points (not 3D network in the whole specimen) and resultant higher strength (shown in Figure 1B). Gamma irradiation was employed, since it is a facile method to control the crosslinking degree. As shown in Figure 1B, after solution casting and hot-press, the specimen will be irradiated with a low dose, followed by multiple-step melting/irradiation (MUSMI) alternately. This strategy exhibits the following advantages relative to conventional irradiation. Firstly, the local crosslinked points are re-distributed during the following melting process, which is an efficient way to avoid high crosslinking density at certain positions and the resultant thermoset property; secondly, it is possible to achieve the relatively high density of crosslinked points via MUSMI, which is the reason for the higher strength; finally, the crosslinked points are expected to act as the heterogeneous nucleation agent during cooling from molten states, producing higher nucleation density, smaller spherulites, and improved ductility.

## 2. Experimental Section

### 2.1. Materials

Poly(vinylidene fluoride) (PVDF, *M*_w_ = 209000 g/mol, *M*_w_/*M*_n_ = 2.0) was purchased from Kureha Chemicals (Tokyo, Japan). Triallyl isocyanate (TAIC) and N, N-dimethylformamide (DMF) were supplied by Sinopharm Chemical Reagent Co., Ltd (Beijing, China). 

### 2.2. Sample Preparation

PVDF/TAIC specimens were prepared by solution casting. PVDF and TAIC (with the weight fractions of 0.5%, 3%, and 10%) were added to DMF and stirred at 80 °C for 3 h to obtain a homogeneous solution with a concentration of 15% (mass fraction). The solution was dried in-oven at 120 °C for 24 h to remove the residual DMF. The dried samples were hot-pressed into films with a thickness of 0.5 mm at 200 °C and 10 MPa. The specimens were vacuum-sealed and then irradiated by γ-ray from a ^60^Co source with the dose of 10 kGy at room temperature (named as 10 kGy*1, shown in Figure 1). Then, the irradiated specimen was heated to 210 °C, followed by hot-press and irradiation for the second time (10 kGy*2). This process was repeated for the third time (10 kGy*3). The reference specimen was irradiated only once at 30 kGy (17 h) after solution casting and hot-press. 

### 2.3. Characterization

A field emission scanning electron microscope (FESEM, Hitachi S-4800, Tokyo, Japan) was used to examine the fracture surface of PVDF/TAIC specimens. The differential scanning calorimeter (DSC, TA, Q2000) was adopted to investigate the thermal behaviors of the specimens. The samples were heated from 30 to 210 °C at a speed of 10 °C/min, held at this temperature for 10 min to erase previous thermal history, and then cooled to 30 °C at a rate of 10 °C/min. The crystallinity (*X*_c_) was computed via Equation (1) [30]:

(1) $$X_{c}=\Delta H_{m}/\Delta H_{m}{}^{\circ}$$

where ∆*H*_m_ is melting enthalpy and ∆*H*_m_° is 290 J/g for the melting enthalpy of perfectly crystalline PVDF. The reaction of TAIC in specimens after γ-ray irradiation was evaluated by Fourier transform infrared spectroscopy (FTIR, Bruker Tensor, Beerlika, MA, USA) with a resolution of 2 cm^–1^. The Instron universal materials testing system (Model 5966) was used for tensile tests at the speed of 10 mm/min. The long periods of PVDF were tested by the small-angle X-ray scattering measurements (SAXS, BL16B1, Shanghai Synchrotron Radiation Facility, China). The wavelength of the monochromatic X-ray beam is 1.24 Å. One dimensional density correlation functions *K*(z) calculated the Fourier transformation of the scattering curve, following Equation (2) [25]:

(2) $$sK\left(z\right)=\left[\overset{\infty }{\underset{0}{\int }}q^{2}I\left(q\right)\cos\left(qz\right)d\left(q\right)\right]/2\pi$$

where *q* is the characteristic wave number, and *I* is scattering intensity. The long periods of PVDF crystals were calculated according to Equation (3):

(3) L=2π/q

The morphologies of PVDF spherulites were observed by a polarizing light microscope (POM, Olympus BX51) with a Linkam LTS 350 hot stage. The samples were heated to 210 °C for 10 min, followed by isothermal crystallization at 150 °C.

## 3. Results and Discussion

**Figure 2:**
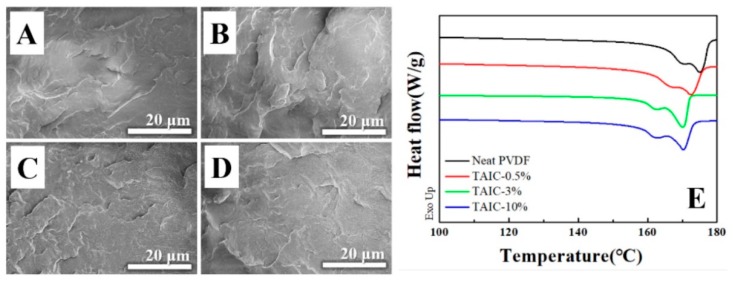
Scanning electron microscopy (SEM) images and differential scanning calorimeter (DSC) curves of neat PVDF (**A** and black curve in **E**) and its blends with various weight fraction of triallyl isocyanate (TAIC, 0.5% in **B**, 3% in **C**, and 10% in **D**) before irradiation.

First of all, it is necessary to assess the thermodynamic miscibility between PVDF and TAIC. For this purpose, the blend specimens with various weight fractions of TAIC (up to 10%) were prepared by solution casting, followed by hot-press. In SEM images (Figure 2A–D) of the fracture surface, there is no obvious aggregation of TAIC. The surface is homogeneous even when the weight fraction of TAIC reaches 10% (Figure 2D). In the DSC curves (Figure 2E), there are two melting peaks located at 169.7 and 175.1 °C in neat PVDF (black curve). Both of these moves to the lower temperature direction upon blending with TAIC (red, green, and blue curves). In the result of TAIC 10%, the values of two melting peaks are 161.9 and 170.1 °C. The remarkable decrease of *T*_m_ indicates that the crystallization of PVDF during cooling was influenced significantly because of the existence of TAIC. This is well known as the “*T*_m_ depression effect” [31]. This result suggests that PVDF and TAIC exhibit excellent miscibility, which has good agreement with the homogeneous distribution of TAIC in PVDF shown in SEM images (Figure 2A to 2D), comparable solubility parameters (25.8 for PVDF and 29.2 for TAIC) [27,28], and the reported results, in which the decrease of melting temperature was also observed [26].

**Figure 3:**
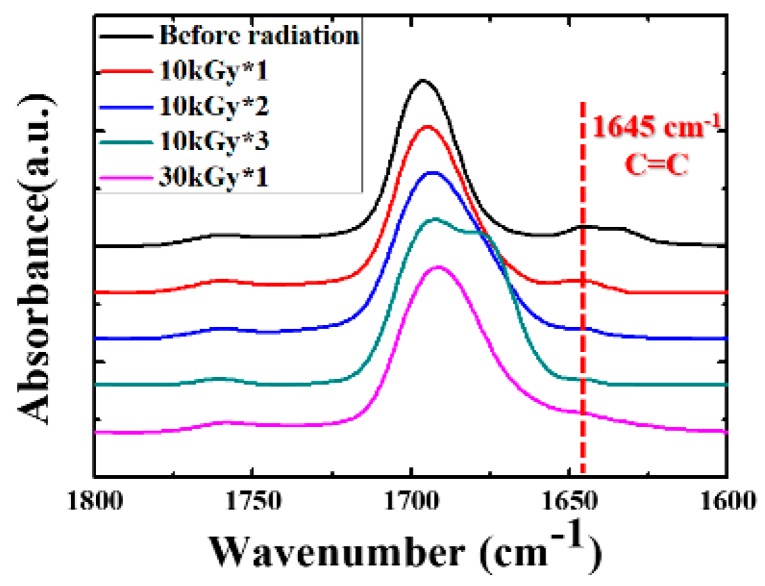
FTIR spectrum of the specimen with PVDF/TAIC (3%) upon irradiation with the indicated dose and times.

Two different irradiation methods were adopted in this work. Specimens were irradiated with the dose of 10 kGy (named as 10 kGy*1), followed by melting at 210 °C, hot-press and irradiation for the second (10 kGy*2) and third (10 kGy*3) time (Figure 1). This is so-called “multiple-step melting/irradiation (MUSMI)”. The specimen irradiated with 30 kGy only once (30 kGy*1) acts as the reference specimen. During irradiation, the radicals can be created in PVDF because of its polarity [32,33], which is the reason for the formation of crosslinked structures in the presence of an agent, e.g., TAIC. In this process, the carbon–carbon double bonds in TAIC participate in the radical reaction. The intensity of carbon–carbon double bonds in FTIR, therefore, is a good parameter to use to describe the reaction. As shown in Figure 3, the absorbance at 1645 cm^–1^, corresponding to the characteristic peak of carbon–carbon double bonds, is obvious before irradiation [26]. It decreases upon MUSMI (from 10 kGy*1 to 10 kGy*3). In the green curve (10 kGy*3), the intensity of this peak exhibits very low magnitude, which is similar with that in the reference specimen (purple curve in Figure 3). The results discussed above clarify that the reaction between PVDF and TAIC was induced by gamma irradiation, accounting for the intensity decrease at 1645 cm^–1^. The comparison of the green and purple curves in Figure 3 indicates that the reaction degrees in the specimen of 10 kGy*3 and the reference are comparable. The structures of these, however, are different. In the former, MUSMI produces local crosslinked points distributed in the whole PVDF matrix. The gel fraction, therefore, is close to zero, corresponding to the thermoplastic properties. The latter suffered from high crosslinking density at certain regions and cannot be dissolved by DMF completely, indicating the disappearance of thermoplastic properties. When the weight fraction of TAIC reaches 5%, the irradiation of 10 kGy*3 also produces thermoset performance. As a result, our discussion focuses on the specimen of PVDF/TAIC (3%) in the following sections.

**Figure 4:**
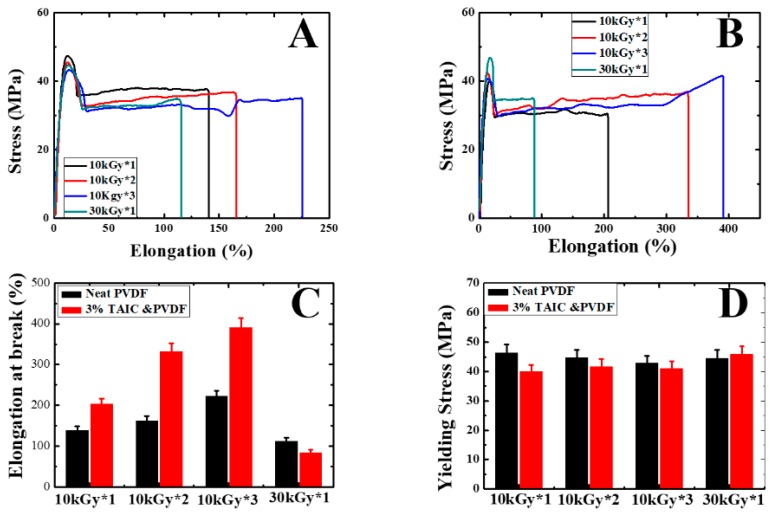
Mechanical performances of neat PVDF (**A**) and PVDF/TAIC (3%, **B**) upon irradiation with the indicated dose and times. **C** and **D** represent the elongation at break and yielding stress, as shown in A and B.

The mechanical performances of neat PVDF and PVDF/TAIC were assessed (Figure 4). In neat PVDF, the film prepared by solution casting and hot-press exhibits a yielding stress of 46 MPa and elongation at break of 138% (data not shown here). The former is not sensitive to gamma irradiation. The latter, however, depends crucially on it (Figure 4A,C,D). The value increases to 140%, 165%, and 226% upon alternate melting/irradiation (10 kGy), for one, two, and three times, respectively. In the case of PVDF blended with TAIC (Figure 4B–D), the yielding stresses of all specimens exhibit similar magnitudes (40–47 MPa, Figure 4D), while the variation of elongation at break becomes more remarkable (Figure 4C). It reaches the maximum of 393% in 10 kGy*3. By contrast, the failure of the reference specimen occurs at an elongation of only 87%. In these results, our attention should be paid to the following issues. Firstly, all specimens (including PVDF and PVDF/TAIC) exhibit similar yielding stress (Figure 4D); secondly, the values of the elongation at break increase upon further melting/irradiation, while they exhibit a lower magnitude in the reference (Figure 4C); finally, MUSMI is a more efficient way to enhance the ductility without the loss of strength relative to strong irradiation. 

**Figure 5:**
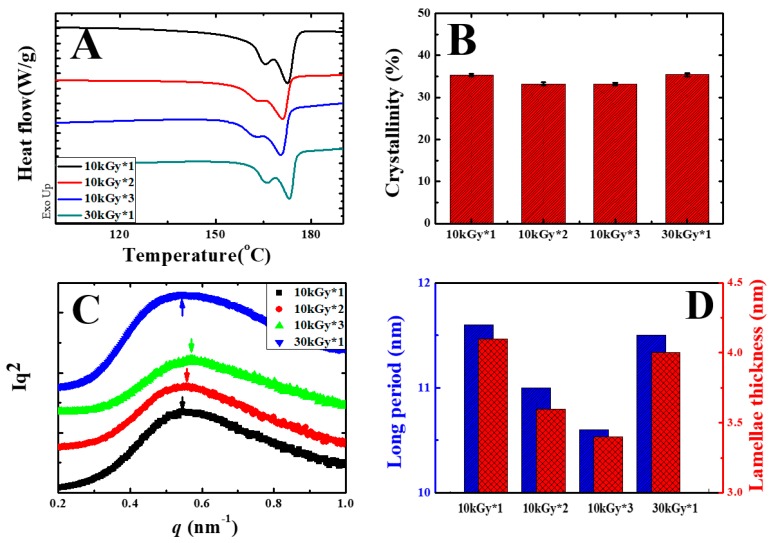
DSC heating curves (**A**) and crystallinity (**B**) obtained from DSC, the Lorentz-corrected SAXS profiles (**C**), and the long period and lamellae thickness (**D**) of cross-linked PVDF films with 3% TAIC after the irradiation with the indicated dose and times.

Both DSC and SAXS were employed to investigate the crystal structures, which play important roles in determining mechanical performance in polymer materials [30,34]. In the first heating curves of DSC (Figure 5A), the double melting peaks of 10 kGy*1 were located at 165.4 and 172.6 °C, both of which move to a lower temperature direction upon further melting/irradiation (red and blue curves). In the specimen of 10 kGy*3, the values are 162.0 and 170.3 °C. In the reference, the melting temperatures are similar with that in the specimen of 10 kGy*1. The reason for the variation of melting temperatures will be discussed in the following parts. The crystallinities of the specimens, calculated according to the DSC curves, are shown in Figure 5B. All of these exhibit close magnitudes ranging from 33.2% to 35.4%. The crystallinities of PVDF, therefore, are not dominating factors of the strength and ductility. In the Lorentz-corrected SAXS profiles (Figure 5C), there are scattering peaks in all specimens. Based on the peak positions emphasized by arrows and one-dimension correlation functions, the long periods and lamellae thicknesses can be calculated. As shown in Figure 5D, both long periods and lamellae thicknesses decrease upon further melting/irradiation. The difference between them, representing the thickness of amorphous parts, remains almost constant (ranging from 7.2 to 7.5 nm). This result indicates that the variation of lamellae thickness dominates the different long periods. Furthermore, the thinner lamellae in Figure 5D accounts for the lower melting temperatures in Figure 5A, and vice versa (crystallinity, lamellae thickness and melting temperatures are listed in Table 1) [35].

**Figure 6:**
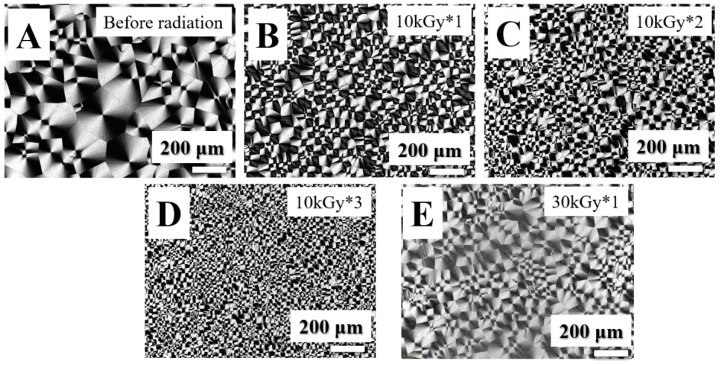
Polarizing light microscope (POM) images of PVDF/TAIC (3%) blends isothermally crystallized at 150 °C completely, upon cooling from melting state before radiation (**A**), 10 kGy*1 (**B**), 10 kGy*2 (**C**), 10 kGy*3 (**D**) and 30 kGy*1 (**E**).

In the following sections, our attention was paid to the PVDF spherulites. The PVDF/TAIC blends (before or after irradiation) were heated to 210 °C (above its equilibrium melting temperature), then cooled down to 150 °C. POM was employed to examine the well-developed spherulites upon isothermal crystallization at this temperature. In the results of the PVDF/TAIC blend before irradiation, the size of spherulites exhibits higher magnitudes, indicating its lower nucleation density (Figure 6A) [36]. When the irradiated specimen was hot-pressed, the subsequent crystallization behavior in the cooling process produced spherulites with smaller diameters (Figure 6B,C). In Figure 6D, there are so many spherulites that it is hard to get the exact size and number of them in the POM images with the current magnification. MUSMI produces the following effects: On one hand, the number of local crosslinked points increases significantly, which can be supported by the intensity decrease of the carbon–carbon double bonds characteristic peak in FTIR (Figure 3); on the other hand, the nucleation density during the cooling process exhibits much higher magnitude, as shown in Figure 6A–D. Obviously, the heterogeneous nucleation effect was enhanced by the crosslinked points resulting from MUSMI, which can be validated by the reference specimen [37]. Relative to MUSMI, the strong irradiation (30 kGy) results in the higher crosslinking density in certain regions. This is the reason for the lower nucleation density and big spherulites during the subsequent cooling process (Figure 6E).

**Figure 7:**
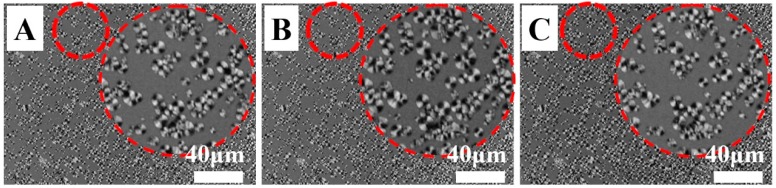
POM images of PVDF/TAIC (3%) blends upon radiation with 10 kGy for three times. (**A**), (**B**) and (**C**) represent the crystallization at 150 °C for 30s from melting state (210 °C) for the first, second, and third time, respectively. The inset parts show the images with higher magnification at the indicated positions.

**Figure 8:**
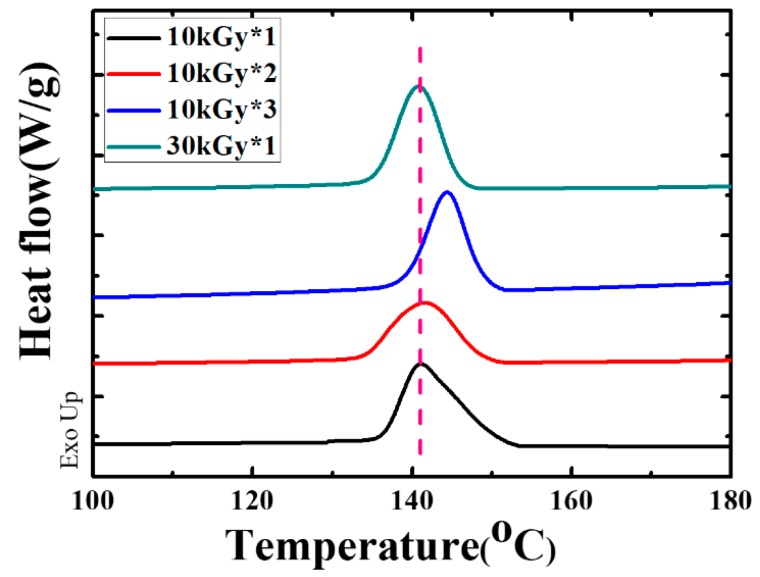
DSC cooling curves upon radiation, with the indicated dose and times.

The heterogeneous nucleation effect was validated further by checking the nucleation position during multiple melting-crystallization and the crystallization temperature (*T*_c_) in the cooling process measured by means of DSC (Figure 7 and Figure 8). On one hand, the specimen of PVDF/TAIC blends upon irradiation with 10 kGy for three times (10 kGy*3) was heated to 210 °C, which was followed by cooling down to 150 °C, and isothermal crystallization for 30 s. The corresponding POM images are shown in Figure 7A. There are many spherulites with diameters of several microns. In the image with higher magnification at the indicated position, the immature spherulites can be observed. After this specimen was melted for the second time, its crystallization behaviors were tracked by POM again. The spherulites occur at exactly the same position (Figure 7B). The same thing happens upon melting/crystallization for the third time (Figure 7C). This result indicates that the crosslinked PVDF undergoes heterogeneous nucleation during cooling. On the other hand, the crystallization temperatures of PVDF upon MUSMI were measured by DSC in the cooling process (Figure 8). In the specimen of 10 kGy*1, the crystallization temperature is at 141.0 °C. This value increases to 141.8 and 144.3 °C in 10 kGy*2 and 10 kGy*3, respectively. The higher crystallization temperature (Figure 8) and nucleation density (Figure 6) suggest that there are extra heterogeneous nucleation agents in the specimens upon MUSMI. Therefore, it is the local crosslinked points that act as the nucleation agent during the crystallization of PVDF, since there are only PVDF and TAIC in this system [36,37]. In the reference specimen, the high crosslinking density in certain regions results in less nucleation points (Figure 6E) and lower crystallization temperature (Figure 8). As a result, its *T*_c_ exhibits a similar value to the specimen of 10 kGy*1, corresponding to the comparable nucleation density and spherulite size, shown in Figure 6B,E.

**Figure 9:**
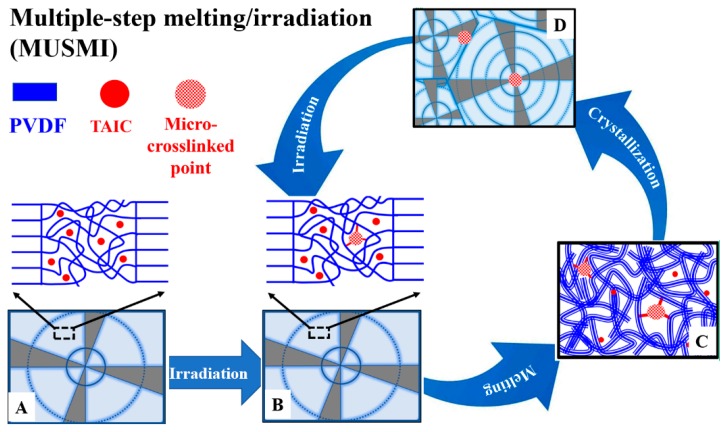
Illustration of PVDF/TAIC blends fabricated by MUSMI: (**A**) PVDF/TAIC blend before irradiation, (**B**) local crosslinked-points appeared upon irradiation, (**C**) re-distribution of crosslinked-points and un-reacted TAIC during melting, (**D**) thinner crystal lamellas and smaller spherulites of PVDF resulted from heterogeneous nucleation effect of local crosslinked points.

According to the discussion above, we can describe the formation of thermoplastic polymers, as well as the enhanced mechanical performance, as follows (Figure 9). PVDF and TAIC exhibit excellent miscibility, which was confirmed by the DSC and SEM results (Figure 1) [27,28,29]. After hot-pressing, PVDF crystallizes, expelling TAIC into the interlamellar regions (Figure 9A) [38]. Upon irradiation for the first time, only a part of TAIC participates in the radical reaction because of the low irradiation dose (10 kGy), producing not 3D networks but some local crosslinked points (Figure 9B) [39,40]. When the specimen is reheated to a temperature above *T*_m_ of PVDF, its crystals collapse, leading to the free diffusion of unreacted TAIC in the molten PVDF matrix. The local crosslinked points can also migrate with the neighboring polymer chains. The melting process, therefore, results in the redistribution of crosslinked points and unreacted TAIC (Figure 9C). The latter participates in the reaction at a “new” position, during the following irradiation. In the process of MUSMI, this redistribution is repeated for several times, contributing to the uniform density of crosslinked points in the whole specimen. This is the reason for the lower gel fraction and thermoplastic properties. The local crosslinked points located in different regions produce remarkable effects on the crystallization behavior during the cooling process and mechanical performance of PVDF. Firstly, some local crosslinked points act as heterogeneous nucleation agents due to the difference of chemical structures with un-crosslinked PVDF matrix (Figure 9D and Figure 7) [41]. This is the reason for the much higher nucleation density, the smaller spherulites (Figure 6), and the elevated crystallization temperatures (Figure 8). Secondly, there are some crosslinked points among the crystal lamellae. The existence of these restricts the lamellar thickening, accounting for the thinner crystal lamellae (Figure 5D) Finally, some crosslinked points distributed in the interspherulitic or interlamellar regions result in enhanced connectivity among spherulites and crystal lamellae. Both thinner crystal lamellae and smaller spherulites endow PVDF with excellent ductility [42]. The better connectivity among crystals is beneficial to the improvement of strength. The synergism of these produces higher ductility without the loss of strength (relative to the reference specimen). The reference specimen suffered from the strong irradiation; however, it exhibited high crosslinking density in certain regions, accounting for the thicker crystal lamellae, lower nucleation density, bigger spherulites, poor ductility (Figure 4B,D), and thermoset properties.

## 4. Conclusions

A multiple-step melting/irradiation (MUSMI) strategy was developed by taking PVDF/TAIC as an example. The alternate melting and irradiation accounts for the redistribution of the local crosslinked points, which is the reason for the thermoplastic properties. During the crystallization of PVDF in the cooling process after melting, the heterogeneous nucleation and restriction effects of these points resulted in smaller spherulites and thinner crystal lamellae, respectively, both of which contribute to the excellent ductility. At the same time, the better connectivity among crystals due to crosslinked points is beneficial to the improvement of strength. As a result of the synergism effect, the prepared PVDF exhibits enhanced ductility without the loss of strength. Our results open up an avenue to fabricate thermoplastic polymers with improved mechanical performance.

## Figures and Tables

**Table 1:** DSC and small-angle X-ray scattering (SAXS) results of cross-linked PVDF films with 3% TAIC upon irradiation, with the indicated dose and times.

Sample	Melting Temperature (°C)	Melting Enthalpy (J/g)	Crystallinity (%)	Long Period (nm)	Lamellae Thickness (nm)
10 kGy*1	172.6	35.9	35.4	11.6	4.10
10 kGy*2	171.1	33.8	33.2	11.0	3.61
10 kGy*3	170.3	33.7	33.2	10.6	3.42
30 kGy*1	172.9	35.8	35.2	11.5	4.02
